# Effects of an Instability Training Program Using Global Versus Selective Instability Devices on Dynamic Balance and Ankle Stability in Young Amateur Soccer Players

**DOI:** 10.3390/jfmk9040240

**Published:** 2024-11-17

**Authors:** Mariana Sánchez-Barbadora, Noemí Moreno-Segura, Vicente Alepuz-Moner, Rodrigo Martín-San Agustín

**Affiliations:** 1 Clinimetry and Technological Development in Therapeutic Exercise Research Group (CLIDET), Department of Physiotherapy, University of Valencia, 46010 Valencia, Spain; mariana.sanchez@universidadeuropea.es (M.S.-B.); valepuz@gmail.com (V.A.-M.); rodrigo.martin@uv.es (R.M.-S.A.); 2 Faculty of Health Sciences, Universidad Europea de Valencia, 46010 Valencia, Spain

**Keywords:** instability, ankle, soccer players, instability devices

## Abstract

**Background/Objectives**: Both Sides Utilized it is one of the most employed global instability devices (GID), but it is difficult to progress and select a particular foot structure. In this sense, the Blackboard has been created as selective instability device (SID). The aim of this study is to compare the effects of both devices on balance and ankle stability. **Methods**: The study was designed as a randomized controlled clinical trial. Twenty healthy amateur soccer players were divided into two groups: GID and SID. Both performed balance training (4-weeks, 3 days/week). Ankle balance and stability were assessed. Paired *t*-tests were used to analyze the pre-, post-, and between-groups differences. **Results**: No differences were found between the groups. Significant intra-group changes were found in both groups for posterolateral balance and summation. Moreover, posteromedial balance increased in the GID group. No changes were found in ankle stability results. **Conclusions**: A balance intervention using GID or SID is effective in improving general and posterolateral balance. Moreover, the GID intervention improved posteromedial balance.

## 1. Introduction

Sport injuries are one of the main concerns of soccer players and their coaching staff [1,2]. Ankle sprains are one of the most common injuries in this sport [3,4] due to the fact that soccer requires sudden stops and pivoting that lead to a situation of muscle and central fatigue [5,6,7]; this results in ankle inversion during plantar flexion, or ankle internal rotation in an inverted position, which most commonly leads to ankle sprain [8]. In addition, sprains have a high recurrence in soccer, leading to pathological laxity, residual pain, and sensorimotor deficits in the ankle, which could cause stability alterations known as chronic ankle instability [3,9]. In this sense, numerous efforts have been made to find alternatives to reduce the incidence and recurrence of ankle sprains. However, the exercise protocols designed to date are still not completely effective since the incidence rates of ankle sprains remain high. Studies that deal with exercises or balance devices that could produce changes in ankle stability and, consequently, reduce injury rates, are still necessary.

The current clinical approach to acute and chronic ankle sprains includes strengthening, balance, and proprioception exercises, such as single -leg stance, restoration of postural control exercises, and coordination tasks [3,4,9,10,11]. These techniques focus on ankle stability training to achieve good dynamic balance. This means the ankle should be able to withstand the presence of forces that would normally alter the state or condition and be capable of returning to an initial state after the disturbance [12]. Maintaining balance is a complex process that not only depends on the integrity of the musculoskeletal and proprioceptive system, but the visual and vestibular systems also play a relevant role [13]. Therefore, therapeutic strategies that take all this into account could be relevant in ankle sprain prevention. 

Previous evidence suggests that muscle strength training programs could improve the activation of the ankle proprioceptors, allowing the automation of balance tasks [10]. It is explained that being strong allows athletes to focus on other aspects of the sport, due to the reduction in central fatigue and, consequently, the risk of injury [10]. Nevertheless, programs that include strategies to improve strength, proprioception, vestibular, and visual components could be an effective alternative given the complete work done on all the components of the balance system. In this sense, training on unstable surfaces has been shown to offer greater benefits than training on stable surfaces [14,15,16,17]. There are many devices that allow for stability training such as Both Sides Utilized (BOSU^®^), balance boards, pads, soft mats, air cushions, or tilting platforms [3,18,19]. Generally, all of these tools are considered global instability devices (GIDs) as the direction and intensity of the instability cannot be selected and adjusted by the user [14].

The most commonly employed GID, having become increasingly popular over the past years, is BOSU^®^. BOSU^®^ combines a solid round base with an inflatable air chamber, and although it highly demands inversion-eversion movements, other movements such as plantarflexion or dorsalflexion could compensate for the lack of balance [14]. However, this device presents some limitations such as the difficulty in progression, the lack of specificity in the foot and ankle structure trained, and the difficulty in portability of the device. In this context, a not yet widely studied device has been designed to overcome these limitations. This is the Blackboard Training, which is a selective instability device (SID) that has demonstrated that it could improve muscle activation of the peroneus longus during single-leg stance, at least as much as other GIDs (including BOSU^®^), finding no differences between devices [20]. These findings may suggest that the use of the SID to improve functional ankle balance in athletes’ ankle sprain preventive programs could be effective, at least as those produced by GIDs, but a clinical comparison has not yet been conducted. If this were the case, the inclusion of Blackboard in ankle instability prevention programs could facilitate the development of the exercises, the progression of the difficulty, the selection of the specific movements worked on, and the portability of the device, making it easier to incorporate this training in multiple environments.

Thus, the aim of this study was to compare the effects of a 4-week balance training program using GIDs or SIDs on functional dynamic balance and functional ankle stability in young healthy amateur soccer players.

## 2. Materials and Methods

### 2.1. Study Design and Participants

The present study was a randomized controlled clinical trial. The randomization process was as follows: of the total number of teams with young soccer players, two teams were randomly selected, one female and one male. Then, a random selection of players who participated in a 4-week balance training program was made within each team. Subsequently, the selected subjects were randomly allocated into two groups: GID balance training (i.e., with a BOSU^®^) and SID balance training (i.e., with Blackboard). The randomization and allocation were performed using the sealed envelope method. Participants were recruited from the Discobolo-La Torre A.C. (Valencia, Spain) with the mediation of the coaches. All participants were informed about the purpose and content of the study and gave their written informed consent to participate in the project. All procedures were approved by the Ethics Committee of the University of Valencia (register number 1236358) and complied with the requirements listed in the 1975 Declaration of Helsinki and its amended version of 2024.

A total number of 20 amateur soccer players made up the study sample. The inclusion criteria were (I) aged between 16 and 35 years, (II) no lower limb surgery during the last year prior to participation, (III) no history of pain in either ankle, knees, or hips during the two months prior to participation, and (IV) not having sprained either ankle for at least three months prior to participation. Those who had participated in lower limb balance and proprioception programs or had suffered balance alterations such as vertigo, vestibular, or central disorders were excluded from the study. 

### 2.2. Procedures

Before the intervention, sociodemographic (age, gender, and dominant leg), anthropometric (height, weight, and leg length), dynamic balance, and ankle stability data were collected. Dynamic balance and ankle stability were also measured at the end of the intervention. To measure leg length, participants were placed in a supine position, and a measurement was taken from the anterior superior iliac spine to the internal malleolus of the same leg.

Firstly, for assessing dynamic balance, the three-direction modified Star Excursion Balance Test (mSEBT) and Emery Test were employed. 

The mSEBT consists of standing on one leg while reaching with the contralateral leg to the farthest possible point in three different directions (anterior, posteromedial, and posterolateral) [21,22]. Adhesive tape was employed to delimit two lines forming a 90-degree angle, and a third line forming a 135-degree angle with respect to the others. Five-millimeter increments were marked on the tape to facilitate measurements [23]. The distance reached in each attempt was normalized with the leg length. Each participant was allowed to make two attempts with each leg and in each direction to practice [24]. Then, three more attempts were performed [25]. A 15-second rest time was allowed between attempts of the same position [23], and five minutes between the different directions [23,24]. All measurements were made barefoot and with hands placed on the hips. For the anterior measurements, the most distal part of the foot was placed at the intersection of the lines, while for the posterior measurements, the heel was aligned with this point [24]. The last three attempts were recorded to calculate the average value. Attempts were discarded in cases of failure to touch the line with the mobile foot, the support foot being moved, hands being released from the hips, loss of balance at some point while resting the mobile foot, failure to maintain the start or end position for at least one second, or supporting the weight on the moving foot at the end of the movement; such attempts were not considered valid and the movement was repeated [23]. The mSEBT has demonstrated excellent inter- and intra-rater reliability [21,26]. 

For the Emery Test, subjects were required to maintain a single-leg stance on an Airex^®^ Balance Pad, with their eyes closed, barefoot, and with their hands placed on their hips [27,28]. The knee of the supporting leg was slightly flexed (at about 30°), and the contralateral knee was at 45° of flexion. The subjects were asked to remain as stable as possible for a maximum time of 180 s [29]. The remaining time of those 180 s minus the time achieved was given as rest time. Three attempts were performed, and the best time obtained was recorded. A handheld stopwatch was used to measure the subjects’ hold position. Before starting the measurements, the subjects were allowed to familiarize themselves with the test for 15 s [28]. When the subjects released their hands from their hips, touched the ground with the contralateral leg, moved the support foot, moved the Airex^®^ Balance Pad from its original position, or opened their eyes, it was considered a loss of balance and the test was repeated [23,28].

Secondly, to assess functional ankle stability, the Side Hop Test was employed to challenge the mediolateral stability of the ankle joint through dynamic inversion and eversion movements, producing a high demand on the peroneus longus muscle [30]. The test consists of jumping laterally on one leg 30 cm delimited by two lines marked on the ground [31,32]. Participants performed 10 repetitions barefoot (a total of 20 jumps) in the shortest possible time. Each participant made three attempts with each leg, with 1-min rests. The best time for each leg was registered. One repetition was allowed before starting for practice. The time was measured with a stopwatch [31,33]. The test was considered failed if the subject touched or crossed the line with their support foot or if the contralateral leg contacted the ground [31].

Finally, a familiarization session identical to the exercise session of the training program was performed. Regarding the intervention, both groups completed a 5-min conventional warm-up, which included stationary cycling and active ankle mobility exercises. Ankle training was then carried out with the different devices (BOSU^®^ or Blackboard). 

**Figure 1:**
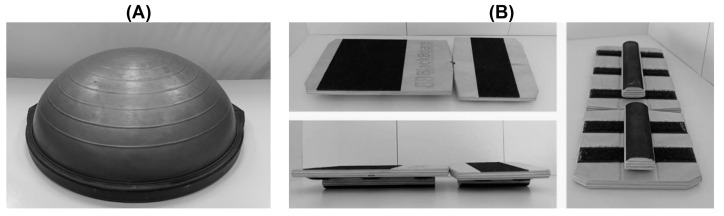
Balance training devices employed. (**A**) BOSU^®^ Balance Training; (**B**) Blackboard Training.

On the one hand, the BOSU^®^ is a device that combines a solid round base with an inflatable air chamber, simulating a split Swiss ball. Both parts can be used to work stability in a different way [14], demanding the inversion-eversion movement. On the other hand, the Blackboard is another device designed for stability training that consists of two wooden boards joined together by a tape. Its base has a Velcro surface where you can freely place half -cylinder wooden slats. Depending on the position where they are placed, one or another type of instability training is generated. Both devices are illustrated in Figure 1. 

The exercise plan was a modified version of the previously proposed plan published by Romero-Franco et al. for proprioception training in athletes [33]. It consisted of four single-leg stance exercises on an unstable surface. All the exercises were performed on the BOSU^®^ in its inverted position or on the Blackboard with the two slats placed centrally (i.e., generating mediolateral instability).

**Figure 2:**
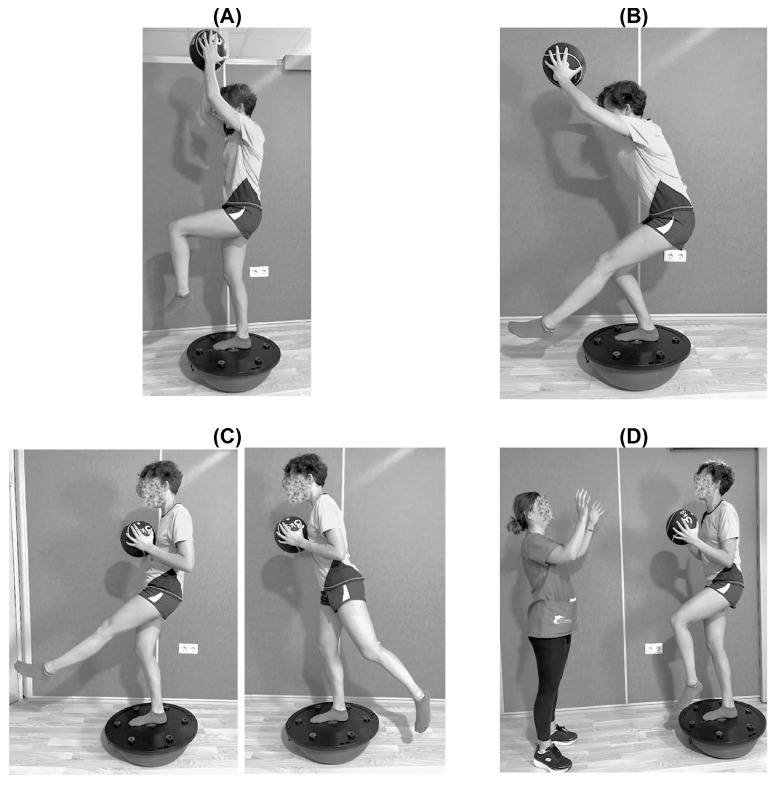
Exercises performed during the balance training sessions. (**A**) Single-leg stance hold. (**B**) Single-leg half-squats. (**C**) Single-leg stance with the contralateral leg balancing from 45° flexion to 45° extension. (**D**) Passes with a partner while maintaining a single-leg stance.

The exercises were the same for both groups and were performed with a 3-kg medicine ball, with the only difference being the unstable surface device [33]. These exercises were as follows: (1) A 30-s series of maintaining the single-leg stance position with an extended knee and hip, holding the ball with the arms stretched out above the head; the free leg was kept at 90° hip and knee flexion. (2) A series of 10 repetitions where the subject was asked to move from the supporting-leg hip and knee starting position to a 90° knee flexion squat, keeping the ball above their head and maintaining the other leg with the knee and hip at about 90° flexion. (3) A single series of 10 repetitions where the subject started with full limb extension of the supporting leg and the ball held with both hands at chest height; from this position, subjects were asked to bring the free leg from 45° hip extension to 45° hip flexion. (4) 10 passes between the subject and a partner where the participants started with a total extension of the supporting limb and the ball held in both hands in front of them at chest height, and their free leg flexed 90° at the hip and knee. A 2-min rest between exercises was allowed. These exercises are illustrated in Figure 2.

The stability training program was performed over a period of four weeks, with three weekly sessions using BOSU^®^ or Blackboard at their soccer club before their usual training and under the supervision of a physical therapist. In total, 12 sessions were completed.

### 2.3. Statistics 

All statistical analyses were carried out using IBM SPSS Statistics software (Version 28.0, IBM Corp., Armonk, NY, USA). Participant characteristics were summarized as means (SD) or frequencies, and stability measures as means (95% confidence intervals (CIs)). The normality of distribution for stability measures was verified using the Shapiro–Wilk test. Unpaired *t*-tests were used to examine the differences in demographic and anthropometric characteristics, and stability measures at baseline between groups. The effects of the balance training program on stability measures were analyzed in separate 2 (Group: SID, GID) × 2 (Time: pre, post) ANOVA with repeated measures on “Time”. The effect size was evaluated with η^2^ (Partial Eta squared), where 0.01 < η^2^ < 0.06 constitutes a small effect, 0.06 < η^2^ < 0.14 a medium effect, and η^2^ > 0.14 a large effect [34]. Where significant main or interaction effects were detected, post hoc *t*-tests with Bonferroni corrections were used (paired for within-group comparisons and unpaired for between-group comparisons). Significance was set at *p* < 0.05 and Cohen’s *d* was also calculated to evaluate the effect size (*d* < 0.2: trivial, 0.2–0.5: small, 0.5–0.8: medium, and >0.8: large) [34].

The sample size was calculated based on a previous study [35], which reported within-group differences for the YBT due to an intervention with an effect size of 1.2. However, to adopt a conservative approach, we estimated our sample size using an effect size of 0.7, with a statistical power of 0.9 and an alpha level of 0.05. This calculation indicated that a minimum of 19 participants was required, which we rounded up to 20 to facilitate group homogeneity.

## 3. Results

A total of 20 participants (9 women and 11 men) made up the final sample. Clinical and demographic variables are depicted in Table 1. No significant differences were found between groups. 

In relation to dynamic balance measured with the mSEBT (Table 2), mSEBT-A showed neither a significant main effect for time nor group-time interaction, while mSEBT-PM, mSEBT—PL, and mSEBT—∑ showed a significant main effect for time (*p* = 0.001; η^2^ =0.54, *p* < 0.001; η^2^ = 0.60, and *p* = 0.001; η^2^ = 0.54) although not for the group × time interaction was observed. Post hoc analyses revealed a significant increase of 0.14 m in mSEBT-PM in both groups and of 0.16 m and 0.21 m in the mSEBT—PL for SID and GID groups, respectively. Regarding mSEBT—∑, it improved 0.13 m in the SID group and 0.10 m in the GID group.

In terms of the results obtained in the Emery Test (Table 2) and the Side Hop Test (Table 3), neither showed a significant main effect for time nor group-time interaction. 

## 4. Discussion

To the best of our knowledge, the present study is the first aimed at comparing the effects of a 4-week (12 sessions) balance training program using GIDs or SIDs on dynamic balance and ankle stability in amateur soccer players. The main findings of this study were that both balance training programs improved several parameters related to dynamic balance without differences between groups, suggesting that both methods could achieve improvements in dynamic balance. By contrast, functional ankle stability did not improve after either of the two interventions.

The most relevant finding is that the equal improvements obtained by both interventions (BOSU^®^ and Blackboard device) in balance could lead to more specific training in athletes. Thus, while the instability produced by the BOSU is indiscriminate across all planes of movement, the Blackboard allows the instability to be directed toward the chosen plane for the objective, in this case, the mediolateral. Consequently, the dynamic balance evaluated by means of the mSEBT improved in both groups, regardless of using GID or SID. This could suggest that instability directed toward inversion-eversion movement could alone be enough to generate adaptations from more global and bulky devices, achieving improvements in dynamic balance. 

Our findings for the GID group are similar to those obtained in previous studies, such as that by Cuğ et al., who carried out a similar BOSU-based 4-week exercise program (12 sessions) in healthy athletes [36]. They also achieved improvements in dynamic balance following their intervention as evaluated by the mSEBT, particularly in the posteromedial- and posterolateral-reach directions of the test. They also found differences in the anterior-reach direction, which did not occur in our case. This may be attributed to the chosen intervention approach, as they directly addressed hopping in their exercises, which could influence performance in the anterior-reach direction of the mSEBT. Nevertheless, based on a previous study where it was shown that the activation of the peroneus longus muscle was similar on the Blackboard as on the BOSU, we decided to focus the intervention on specific mediolateral plane instability. Thus, to our knowledge, this is the first time that the effects of an SID intervention on dynamic balance in an athletic population have been studied. Since dynamic balance improved similarly in both groups of amateur soccer players, we consider that our proposal is an interesting starting point, and future studies could apply this program for injury prevention, mainly ankle sprains, or address the potential benefits of including SID training in rehabilitation schemes for chronic or acute ankle instability.

In terms of functional ankle stability, unexpectedly, no changes were observed in either of the groups. While ankle stability is multifactorial, we expected that instability training would have a positive effect on it, but this was not the case. This may be due to a ceiling effect since all the participants were young, sporty, and healthy subjects and, therefore, the probability of improvement is low. In this regard, research by Linens et al. found a cut-off score of 12.88 that discriminates between people with and without postural instability, and in our sample, the mean values at baseline were 10.05 for the SID group and 9.63 for the GID group; accordingly, when a population group is already at optimal values, obtaining a significant improvement will be more complex [37].

On the other hand, this study shows that an improvement in balance does not always imply an improvement in ankle stability. This may be explained by the fact that balance relates to the ability to maintain a stable and controlled posture, while ankle stability refers specifically to the ability of the ankle joint to resist excessive movement and avoid injury. Therefore, the exercise program implemented in the present study may be more focused on improving general balance than on improving stability, where other components such as strength, proprioception, specific neuromuscular control of muscles and ligaments, coordination, and movement technique, may enjoy greater benefits. Therefore, given the multifactorial origin of ankle stability, an approach focused on an analysis of the athlete’s specific deficiencies could be more effective than a generalized protocol, to obtain significant improvements in healthy athletes.

### 4.1. Implications for Practice and Research

Our study has important clinical applications. Our findings suggest a possible beneficial use of this exercise program with BOSU^®^ or Blackboard to preventively improve dynamic balance in healthy young amateur soccer players, which could potentially reduce their risk of ankle sprains. Additionally, considering our findings with similar improvements for both GID- and SID-based balance programs, this could imply that the Blackboard could serve as an alternative to the BOSU^®^, which is the most commonly used GID within the sports field, as the chosen instability device in these programs. For this reason, since the Blackboard is more portable and smaller than the BOSU^®^, it could facilitate its use and implementation, allowing athletes to use it regularly within their sports practice. On the other hand, the Blackboard as an SID allows for a wide range of instability settings, being able to configure its direction and intensity depending on how the slats are placed. Thus, this study promotes the implementation of a program and tool that can be used in future studies using different configurations or applied to different populations.

### 4.2. Strengths and Limitations of the Study

This study had several strengths. First, our experimental protocol was similar to previous work, which facilitated comparison [33]. In addition, the tests used to measure the changes produced by the intervention have been widely used in the literature. Second, we are the first to propose a program using SID to improve the balance of amateur soccer players. Since SIDs have the advantage of being more portable, cheaper, and configurable than GIDs, we consider that multiple athletes can benefit from the use of this type of instability device. Moreover, it could even be used as a device for autonomous training of the athlete due to its portability.

Despite the relevant findings, this study is not without limitations. First, the population studied included only healthy young amateur soccer players; therefore, the results cannot be extrapolated to a pathological population or to other sports. as previously mentioned, obtaining significant improvement was challenging. Secondly, only two instability devices were employed, and other devices may have been more demanding in terms of balance and ankle stability, but this was outside the objectives of the study, as the aim was to compare the effectiveness of a relatively new SID such as the Blackboard with the most used GID, the BOSU^®^. Thirdly, due to the nature of the study design, the low availability of the soccer players (given that they were not professional teams and because of their work schedules), the available sample size has been small. Therefore, future studies with a larger sample size should be carried out. Fourth, the exercise program proved to be not very demanding in producing adaptations in ankle stability in healthy subjects, which might have been different if hopping exercises had been included in the intervention. Fifth, the study includes soccer players of both genders who continued their regular training alongside the intervention, which could induce adaptations in balance too and make it difficult to analyze the isolated effects of the interventions. future studies should include a control group. Considering the above and having demonstrated the effectiveness and safety in healthy individuals, it could be interesting to apply this protocol in the recovery of subacute sprains and chronic ankle instability to analyze the results in pathological amateur soccer players, where improvements in ankle stability could be higher.

## 5. Conclusions

In conclusion, according to the results obtained from healthy amateur soccer players, a balance intervention using either the BOSU^®^ or the Blackboard unstable surfaces is effective for improving general and posterolateral balance. Moreover, BOSU^®^ intervention improved posteromedial balance. By contrast, there were no differences between groups and no changes in ankle stability after either intervention. Thus, subject to further research, this type of intervention could be beneficial in ankle injury prevention and sprain rehabilitation programs for amateur soccer players, especially the Blackboard, which could be implemented on soccer fields and in installations due to its easy portability. Moreover, both interventions could be implemented in gym programs to improve ankle balance and stability, extrapolating these results not only to soccer players but also to other types of athletes.

## Figures and Tables

**Table 1:** Descriptive characteristics of the participants.

	SID Group (*n* = 10)	GID Group (*n* = 10)
Age (years)	23.0 (5.68)	21.0 (3.80)
Sex (women/men)	5/5	4/6
Height (cm)	169.70 (8.89)	168.17 (5.25)
Weight (kg)	73.80 (18.34)	65.89 (6.79)
Dominant leg (right/left)	5/5	9/1
Right leg length (cm)	88.40 (4.70)	87.39 (3.05)
Left leg length (cm)	89.10 (4.64)	88.11 (3.19)

Data are expressed as mean (standard deviation); mm: millimeters; kg: kilograms. SID: selective instability device; GID: global instability device.

**Table 2:** Differences within and between groups for dynamic balance measured using the mSEBT and Emery Test.

	SID Group (*n* = 10)			GID Group (*n* = 10)			SID-GID
	PRE	POST	*p* (*d*) *	PRE	POST	*p* (*d*) *	*p (*η^2^*)*
mSEBT – A	0.69; 0.65–073 (0.02)	0.72; 0.63–0.82 (0.04)	0.889	0.72; 0.63–0.81 (0.04)	0.72; 0.64–0.80 (0.04)	0.484	0.389 (0.05)
mSEBT—PM	0.92; 0.80–1.04 (0.05)	1.06; 0.99–1.12 (0.03)	0.013 (3.40)	0.96; 0.85–1.07 (0.05)	1.10; 0.97–1.22 (0.05)	0.013 (−2.80) *	0.987 (0.01)
mSEBT—PL	1.06; 0.96–1.16 (0.04)	1.22; 1.11–1.34 (0.05)	0.014 (−3.53) *	1.06; 0.93–1.19 (0.06)	1.27; 1.16–1.38 (0.05)	0.003 (−3.80) *	0.564 (0.02)
mSEBT—∑	0.90; 0.81–0.99 (0.04)	1.03; 0.96–1.1 (0.03)	0.006 (−3.68) *	0.90; 0.83–0.96 (0.03)	0.99; 0.92–1.07 (0.03)	0.024 (−3.00) *	0.629 (0.02)
EMERY TEST	5.47; 2.05–8.9 (1.45)	11.44; 4.02–18.86 (3.14)	0.093	5.76; 3.95–7.58 (0.74)	5.03; 3.93–6.13 (0.45)	0.327	0.094 (0.19)

Data are expressed as mean; 95% confidence interval (standard deviation). mSEBT: three-directions modified Star Excursion Balance Test; SID: selective instability device; GID: global instability device. mSEBT-A: three-direction modified Star Excursion Balance Test anterior; mSEBT-PM: three-direction modified Star Excursion Balance Test posteromedial; mSEBT-PL: three-direction modified Star Excursion Balance Test posterolateral; mSEBT—∑: three-direction modified Star Excursion Balance Test Summation. *n* = sample size; *p* = *p*-value; *d* = Cohen’s *d*; η² = partial eta squared; * = significant change.

**Table 3:** Differences within and between groups for ankle stability measured using the Side Hop Test.

	SID Group (*n* = 10)			GID Group (*n* = 10)			SID-GID
	PRE	POST	*p*	PRE	POST	*p*	*p* (η^2^)
SIDE HOP TEST	10.05; 7.87–12.23 (0.92)	9.17; 6.43–11.91 (1.16)	0.292	9.63; 7.8–11.46 (0.75)	8.96; 7.1–10.82 (0.76)	0.212	0.827 (0.01)

Data are expressed as mean; 95% confidence interval (standard deviation). SID: selective instability device; GID: global instability device. *n* = sample size; *p* = *p*-value; *d* = Cohen’s *d*; η² = partial eta squared.

## Data Availability

The raw data supporting the conclusions of this article will be made available by the authors on request.
